# Prevalence, intensity and factors associated with soil-transmitted helminths infections among preschool-age children in Hoima district, rural western Uganda

**DOI:** 10.1186/s12879-018-3289-0

**Published:** 2018-08-17

**Authors:** Silvestro Ojja, Stevens Kisaka, Michael Ediau, Doreen Tuhebwe, Angela N. Kisakye, Abdullah A. Halage, Richard K. Mugambe, Joan N. Mutyoba

**Affiliations:** 1 Department of Biostatistics and Epidemiology, Makerere University School of Public Health, Kampala, Uganda; 2 Department of Health Policy Planning and Management, Makerere University School of Public Health, Kampala, Uganda; 3 Department of Disease Control and Environmental Health, Makerere University School of Public Health, Kampala, Uganda

**Keywords:** Preschool-age children, Soil transmitted helminths, Intensity, Hoima

## Abstract

**Background:**

Over 80% of morbidity due to soil-transmitted helminthiasis (STH) occurs in low-income countries. Children under 5 account for 20–30% of the burden in endemic areas. This study assessed the prevalence, intensity and factors associated with STH infections among preschool-age children (PSAC) in Hoima district, Uganda. The PSAC are particularly vulnerable because the chronicity of this condition usually affects their physical and mental growth and development.

**Methods:**

A cross-sectional study was carried out among 562 PSAC (1–5 years old) in 6 counties of Hoima district using Expanded Program on Immunization (EPI) method. Stool samples from children were examined using the formol ether concentration technique for STH egg detection. Egg counts were represented as egg per gram (EPG). A structured questionnaire was used to collect information on factors associated with STH infection. Generalized linear models were used to analyze relationships between STH infection and associated factors.

**Results:**

Overall STH prevalence was 26.5%. Hookworm infection was the most prevalent (18.5%), followed by *A.lumbricoides* (9.8%) and *T.trichiura* (0.5%). Prevalence of STH infection was significantly higher in children aged 5 years (Pearson chi-square test, *p* = 0.009) than in children aged 1 year. The general geometric mean (GM) counts for Hookworm infection was (696.1 EPG; range (530.3–913.8)) with girls having a higher GM (789.8 EPG; range (120–13,200)) than boys. Eating uncooked or unwashed vegetables (adj. Prevalence Ratio (PR) = 1.9, 95% CI: 1.3–2.7) and fruits (adj.PR = 1.8, 95% CI: 1.1–2.8), indiscriminate disposal of young children’s faeces (adj.PR = 1.5, 95% CI: 1.1–2.0); not washing hands after defecation (adj.PR = 2.6, 95% CI: 1.9–3.6); and not deworming children regularly (adj.PR = 1.4, 95% CI: 1.1–1.8) were significantly associated with STH infection.

**Conclusion:**

The prevalence of Soil transmitted helminths infection among preschool-age children in Hoima district significantly increased with age. Poor hygiene, inadequate sanitation and irregular deworming were associated with STH infections among PSAC in the study area. Intense health education on the importance of hygienic practices, improved sanitation and regular deworming of PSAC should be integrated into prevention and control programs.

**Electronic supplementary material:**

The online version of this article (10.1186/s12879-018-3289-0) contains supplementary material, which is available to authorized users.

## Background

Soil transmitted helminthes (STH), such as Ascaris lumbricoides (Round worm), Trichuris trichiura (Whipworm) and Ancylostoma spp (hookworms) are nematodes that infect more than a billion people worldwide, with low-income countries accounting for over 80% of the disease burden [1–3]. In areas with inadequate water, sanitation and hygiene conditions, children are particularly at higher risk for infections due to STH [4–7]. The affected children are usually malnourished and anemic because of the resultant nutritional deficiency [8, 9]. Long term effects associated with the condition include diminished physical fitness, growth retardation and delayed intellectual development and cognition [10, 11].

Some studies have associated STH infections with poverty, poor nutrition, nutritional insecurity, poor sanitation, lack of clean drinking water and low deworming uptake [9, 12–18]. Many of these studies however, focused on the school children on the basis of easy reach [15]. These studies also did not examine the influence of education, access to health services and household socio-economic status on the burden of STH in children [15].

Uganda started implementing the national child day plus in 2004 [8, 19]. The strategy targets children aged 1–14 years old and it was adopted to treat Schistosomiasis and STH with praziquantel and Albendazole. The aim is to reduce prevalence and intensity of STH and the risk of long term effect of STH infections in children. However, this approach misses out many preschool-age children whose caretakers fail to attend child day plus due to poor health seeking behaviours [1, 9, 20, 21]. Even where data has been available, it shows low deworming coverage in the country [22].

In Hoima district, deworming treatment coverage for STH was estimated at 60% in 2015, which was well below the national target of 80% and the prevalence of STH in the district was estimated at 22% [23, 24]. Preschool-age children accounted for 9% of the overall 22% STH prevalence in the district, despite the existence of the child day plus which has been ongoing since 2004 [24]. The most affected sub-counties in the district include: Kyangwali, Kabwoga, Buseruka, and Kigorobya and areas around the shores of Lake Albert where sanitation and hygiene conditions are poor [6, 24]. There is inadequate information on the prevalence, intensity and factors associated with STH infection among preschool-age children (PSAC) in Hoima district. This study therefore aimed at determining the prevalence, intensity and the factors associated with STH infections among PSAC in Hoima district so as to provide basis for appropriate strategies against STH infections.

## Methods

### Study setting and design

This was a cross-sectional study in which a structured questionnaire was used to collect data from caretakers of the preschool-age children aged 1–5 years old in 51 villages in Hoima district from March to April 2016. Hoima district is located in the western region, about 220 km from the capital city Kampala, Uganda. The district is made up of 13 sub-counties, 54 Parishes and a total of 643 villages with an estimated total population of 572,986 people. Over 90% of the population lives in rural areas and over 70% are peasant farmers who depend on subsistent agriculture for their livelihood needs. In Hoima, STH is more prevalent in lowland areas and areas along the shores of Lake Albert. Our study was conducted in March 2016 prior to the planned national deworming exercise.

### Study population

The study participants comprised of 562 preschool-age children 1–5 years old selected from villages of 12 Parishes in Hoima district.

### Sample size determination and sampling procedure

The sample size for this survey was estimated using Bennett’s cluster survey sampling formula estimating prevalence of 22% and assuming a precision of 0.03 type 1 error of 5%, design effect of 1.9, intra-cluster variability of 0.1 and number of respondents per cluster as 10 [25, 26], a sample size of 510 was obtained. Assuming 10% non-response rate, we sampled 561 participants to cater for non response. We actually collected data from 562 participants, after additionally identifying two preschool-age children from adjacent homes with diarrheal conditions.

The 2015 annual district Water Sanitation and Hygiene (WASH) report revealed that 11 Parishes in 4 lowland sub-counties of Kyangwali, Buseruka, Kabwoya and Buhimba and 9 Parishes in 2 upland sub-counties of Bugambe and Kiziranfumbi had poor sanitation and hygiene conditions. Therefore, on the basis of this background information, we selected a total of 12 Parishes (6 Parishes each from lowland and upland areas) using simple random selection method (balloting) without replacement. A total of 51 villages were selected from the 12 selected Parishes with probably proportionate to size of village in each Parish. We recruited 562 participants, one per household in the selected villages. To achieve this, the total number of households in each village was obtained from the Local Chairperson (LC-1) and a sampling interval K was calculated by dividing the total number of households per village by the number of household targeted for inclusion in the study in order to get the required number of target respondents per village. The research team located the center of the chosen village with the help of the LC-1 or representative and randomly selected a direction by using a compass to determine the starting direction. The team then moved in the northern direction as indicated on the compass to locate the starting household from which an eligible child aged 1–5 years old was selected to participate in the survey. In a household with more than one child aged 1–5 years old, one was recruited in the survey using simple random selection method without replacement. A household that did not have a child who met the inclusion criteria was substituted with the next household. The data collectors then moved every K^th^ household in clockwise concentric circles looking for eligible child till the required sample for that village was obtained. Respondents were caretakers of children aged between 1 and 5 years of age and have lived in Hoima for at least one year. Preschool-age children who were sick, who failed to defecate, who received deworming in the last 2–3 months or whose caretakers were absent at the time of the survey were excluded. Respondents of the PSAC were asked to tell the ages of their children in completed years and where possible, trained research assistants verified ages with immunization cards and/or birth certificates.

### Data collection and laboratory procedure

Caretakers of the participating children were oriented on specimen collection procedures prior to specimen collection. Plastic Stool containers with applicator sticks were then marked with identification numbers and distributed to the caretakers of each selected child to submit about 5 g (a size of a thumb) of fresh stool samples, which were collected in the morning and examined in the afternoon of the same day. The stool samples and data collected using structured questionnaire for each child was assigned similar codes. The team made follow-up visit to collect samples from eligible children who failed to defecate on the date of the survey.

On the same day of stool collection, a structured questionnaire was administered to children’s caretakers so as to obtain information on Socio-demographic, economic, behavioural and community factors, as well as knowledge and practices about STH infections. The questionnaire was adopted from standard tools provided by the joint monitoring programme (JMP) of World Health Organization (WHO) and United Nations International Children Education Funds (UNICEF) [27]. The questionnaire was pre-tested and suitably modified through a pilot study in Kigorobya village that was not included in the sample.

Stool samples were stored in properly sealed boxes and transported to Hoima Regional Referral Hospital for analysis in the afternoon of the same day. The Formol- ether-sedimentation technique was used to separate the parasite from fecal debris, and unmask for visibility [28, 29]. The fecal samples were examined for appearance, colour, blood, and mucus.

In summary, an aliquot (matchstick head amount) of stool for each child was picked using applicator stick and mixed with formol water contained in screwed tube and mixed by shaking for at least 30 s. The floating liquid on top of the sediment was filtered through a sieve into a second centrifuge tube. After adding 3–4 ml (ml) of ethyl acetate, the sentiment in the second tube was centrifuged at minimum speed (2500 revolution per minute (rpm)) for 5 min [30]. The final sediment was then used for slide preparation. Thick smears were examined microscopically in the same way as that of the direct saline method [30]. The number of helminths eggs were counted and recorded for each species separately. To increase chances of diagnosing STH infection, 2 slides of the same stool sample were prepared and examined by two experienced laboratory technicians and for any discrepancies in diagnosis, the technicians re-examined stools inter-changeably to determine correct STH infection.

Data was collected on, socio-demographic, economic, behavioural and community factors, as well as knowledge and practices about STH infections. The primary outcome variable was STH infection, defined as any child aged 1–5 years in whose stool sample at least one type of soil helminthes parasite has been identified according to laboratory results. STH infection was measured as either ‘yes’ (infection present) or ‘no’ (no infection). Where the parasite was present, secondary outcome variable (the intensity of infection) was also measured as the number of soil helminthes eggs per gram of faeces [31]. Different thresholds of parasites according to this criterion included: *A. lumbricoides* (Round worms) (1–4999 egg per gram (EPG) = light intensity), (5000–49999EPG = moderate intensity) and (> = 50,000 = heavy intensity); *Ancylostoma spp (*Hookworms) (1–1999 EPG = light intensity, 2000–3999 EPG = moderate intensity, > = 4000 EPG = heavy intensity); *T. trichiura* (Whipworm) (1–999 EPG = light intensity, 1000–9999 EPG = moderate intensity, > = 10,000 heavy intensity).The number of eggs per gram of faeces was calculated by adding the egg counts in all fields together then multiply the total by 24 to give the EPG of faeces. (Example: 15 eggs are seen in total, then 15 * 24 = 600 EPG (WHO, 1987, 1993).

Data on socio-demographic characteristics was collected based on age, gender, residence, education, occupation, religion, and birth order. To classify socio-economic status (SES), wealth index was computed using, principal component analysis (PCA) based on ownership of 14 household assets including: 1) television; 2) radio; 3) mobile phone; 4) bicycle; 5) motorcycle; 6) moto vehicle; 7) piece of land; 8) large farm animals like cattle, goats and sheep; 9) small farm animals like poultry; 10) Machine made wooden/metallic bed; and 11) house wall made of brick; 12) house wall made of mud; 13) house floor made of cement and 14) house floor made of bare-earth floor as earlier described [32, 33]. Each household item was then assigned a weight or factor score generated through principal components analysis. The resulting asset scores were then standardized and the component on which factor loading appeared the most was used to generate total score for each respondent. The respondents’ scores was then grouped into quintile of wealth index (Lowest, second, middle, Fourth and Highest) using the PCA [34]. 20% of the population with lowest total asset scores became individuals in the lowest wealth quintile, the next 20% were members of the second quintile, up to the highest quintile [34]. The same approach was used by other studies and Demographic Surveillance Surveys in Africa [32, 33].

Knowledge about STH infection was measured in four domains including: mode of transmission/causes; prevention; choice of treatment option as well as; schedules for the annual mass deworming program. Participants were scored with a 1 for each knowledge items they knew, and with a 0 for those they did not know. The total score for the 17 questions used in the assessment of knowledge were used to grade participants into four knowledge levels: very low (0–4), low (5–9), moderate (10–14) and good (15–17). Participants in the ‘moderate’ and ‘good’ categories were regarded as having adequate knowledge about STH infections [35]. Behavioural factors: sandal/shoe wearing (not at all, sometimes, always), child eating habits (communal, self services, fed by someone else), frequency with which a child eats away from home (Not at all, Sometimes, often times), meal preparation (person aged < 15 years, Person aged 15–19 years, person aged 20+ years), child eating vegetables without washing /cooking (not at all, Sometimes, often times/always) and child eating fruits without washing them (not at all, Sometimes, often times/always) and community factors: household latrine facilities (open defecation, pit latrine, water closet). Sources of drinking water (safe, unsafe), disposal of young child’s feces (sanitary, unsanitary), disposal of domestic waste (sanitary, unsanitary), child received de-worming treatment in April 2015 (Yes, No), and child received de-worming treatment in October 2015 (Yes, No) were measured using nominal and dichotomous scales. In this study, sanitary disposal of human and animal faeces was defined as putting the faeces in the latrine or burring it into the soil [36].

### Statistical analysis

Data was double entered into Epi-info version 3.5.1, cleaned and exported to STATA12 (StataCorp.; College Station, TX, United States of America) for analysis. Data exploration and reduction was undertaken and independent variables were selected and analyzed to determine their associations with the outcome variables. For continuous variables, summary statistics was obtained including means (±Standard Deviation (SD)). For categorical variables, proportions and frequencies were computed. Prevalence of STH infections and STH infection intensity disaggregated by sex, age group and residence were cross-tabulated and *p*-values computed using Persons chi-square test as well as t test for mean age of the PSAC. Homogeneity of variance among groups such as sex, residence and age group were also tested using Bartlett’s test.

Prevalence ratios (PRs) were used as measure of association between the outcome variables and the independent factors. They were computed using a generalized linear model (GLM) analysis with Poisson family and a log link with robust standard errors. All independent factors with *p*-value < 0.15 [37] and biologically plausible variables were included in the multivariable model. Logical model building technique was used to ascertain the best fitting model with a log likelihood tending towards zero. For all tests, a p-value ≤0.05 at 95% confidence intervals (95% CIs) were considered statistically significant. Unadjusted (PRs), adjusted prevalence ratios (adj.PR), and their respective 95% CI were reported.

## Results

### Demographic characteristics of preschool-age children

A total of 562 preschool-age children participated in the study and all their caretakers were interviewed, giving a 100% response rate. The response rate was 99.1% (557/562). The reasons for not obtaining 100% response rate included: 1) absence of caretakers at the time of stool sample collection, 2) some children failed to defecate in the morning, and 3) some caretakers misplaced stool containers. The mean age of the participating children was 3.2 years (SD = ±1.3). Majority 78.5% (441/562) of the children were aged 3–4 years. There were more girls 53.9% (303/562), *p* = 0.0157 than boys. Slightly more than half 51.1% (287/562) of the children lived in upland areas. There were no significant sex and age differences between residence [Bartlett’s test for equal variances: chi-square = 0.003, d.f (1, 560), *p* = 0.960 and chi-square = 0.0043, d.f (2, 559), *p* = 0.798) respectively. The rest of the results are shown in Table 1 below.

**Table 1:** Demographic characteristics of the 562 preschool-age children that participated in the study in Hoima district, rural western Uganda

Variable	Frequency (*N* = 562)	Percentage (%)
Gender		
Male	259	46.1
Female	303	53.9
Age in completed year		
1–2	172	30.6
3–4	269	47.9
5	121	21.5
Birth order		
1	124	22.1
2	120	21.4
3	91	16.2
4	60	10.7
> 4	167	29.6
Location of residence		
Lowland area	275	48.9
Upland area	287	51.1

### Socio-demographic and economic characteristics of the caretakers

Among the caretakers who provided information on behalf of their children; more than half 54.3% (305/562) were aged 30–39 years. Majority 62.5% (351/562) of the caretakers were married and 63.7% (221/562) were Christians. About 45.0% (246/562) of the caretakers attained primary education, whereas 7.3% (41/562) had tertiary education. The difference in education of caretakers across residence was not statistically significant (Bartlett’s test for equal variances: chi-square = 0.0930, d.f (3, 558), *p* = 0.993). Almost three-quarters 74.0% (416/562) of the participants were peasants (Table 2).

**Table 2:** Socio-demographic and economic characteristics of caretaker of the 562 preschool-age children that participated in the study in Hoima District

Variable	Frequency (N = 562)	Percentage (%)
Number of children under five		
1	176	31.3
2	261	46.4
3	83	14.8
> 3	42	7.5
Marital status		
Never married	152	27.0
Married	351	62.5
Widowed/divorced	59	10.5
Caretaker’s education level		
No formal education	144	25.6
Primary	227	43.8
Secondary	150	23.3
Tertiary	41	7.3
Caretaker’s occupation		
Peasant	413	73.5
Self employed	111	19.7
Formal employment	38	6.8
SES quintiles		
Lowest	114	20.3
Second	111	19.8
Middle	122	21.7
Fourth	107	19.0
Highest	108	19.2
Religion		
Christians	358	63.7
Non Christians	204	36.3

### Prevalence of soil transmitted helminths infections among preschool-age children

Out of 562 children whose stool samples were examined, 26.5% (149/562) were positive of STH infection, while 73.5% (413/562) did not show any evidence of infection. Parasite specific infection prevalences were 18.5% (104/562) for Hookworm, 9.8% (55/562) for *A. lumbricoides* and 0.5% (03/562) for *T. trichiura*. Hookworm and *A. lumbricoides infections* were reported in all the age groups. The prevalence of STH infections was significantly higher in boys than in girl. This difference was statistically significant (Pearson chi-square test, *p* = 0.002). Children living in lowland residential areas presented with higher prevalence of Hookworm and *A. lumbricoides infections* compared to children living in upland residential areas (*p* = 0.009, and *p* = 0.002) (Additional file 1).

**Fig. 1:**
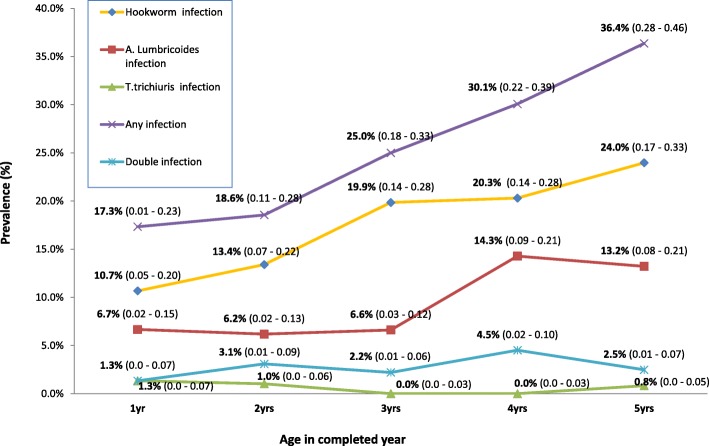
Age disaggregated distribution of intestinal helminths among the 562 preschool-age children that participated in the study in Hoima District

**Fig. 2:**
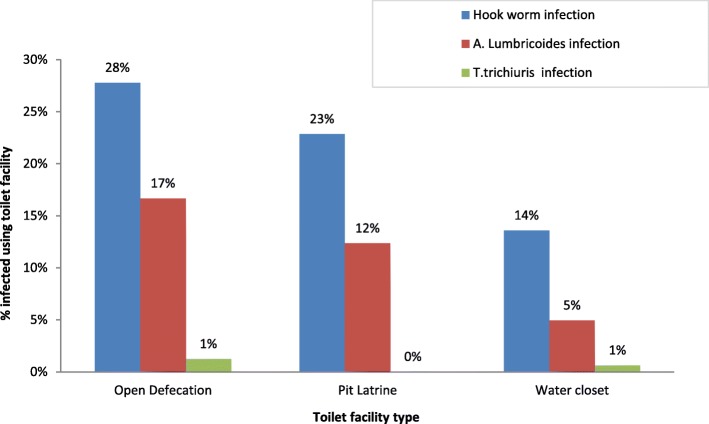
Distribution of intestinal helminths among the 562 PSAC influenced by sanitary facilities in Hoima District

Prevalence of Hookworm infection was significantly higher in children aged 5 years (Pearson chi-square test, *p* = 0.045) than in children aged 1 year. There was significant sex difference in the prevalence of Hookworm infection (Pearson chi-square test: *p* = 0.004), but insignificant sex difference was observed in the prevalence of *A. lumbricoides and T. trichiura* infections (Pearson chi-square test: *p* = 0.298 and *p* = 0.657). The prevalence of Hookworm and *A. lumbricoides* coinfections was 2.8% (16/562). The 4 year old children exhibited the highest 4.5% (6/133) infection prevalence of (Hookworm + *A. lumbricoides*) co-infection, compared to other age groups (Additional file 1). However, these differences were not statistically significant (Pearson chi-square test, *p* = 0.693). Children aged 3–4 years had no *T. trichiura* infection. None of the children were diagnosed with multiple STH (Fig. 1, Additional file 2). Hookworm and A.*lumbricoides* infections were more prevalent 22.9% (95%CI: 0.18–0.28) among children living in homes where household members defecated in the open (Pearson chi-square test: *p* < 0.001) (Fig. 2, Additional file 3).

### Intensity of soil transmitted helminths among preschool-age children

The geometric mean faecal egg count of Hookworm among infected children was 696.1 EPG (range: 48–13,200). For *A. lumbricoides,* it was 569.7 EPG (range: 120–7200) and for *T. trichiura* the count was 440.0 EPG (range: 168–1920). The geometric mean faecal egg count of Hookworm among children aged 5 years was 936.9 EPG, (95% CI: 562.3–1560.9) significantly higher compared to 482.9 EPG (95% CI: 281.6–828.3) and 689.3 EPG (95% CI: 456.4–1041.0) among children aged 1–2 and 3–4 years, respectively (F = 4.36, *p* = 0.012). Children infected with *A.* lumbricoides had the highest 663.6 EPG (95% CI: 436.5–1009.0) compared to the other age groups. However, this difference was statistically insignificant (F = 0.4, *p* = 0.668). Similarly there was insignificant age difference in the *T. trichiura* infection among PSAC (F = 1.37, *p* = 0,254). Females had the highest geometric mean faecal egg counts of hookworm 789.8 EPG (95% CI: 511.6–1219.3), compared to the mean faecal egg counts of 539.0 EPG (95% CI: 446.9–913.6) for males (*t* = − 3.21, *p* = 0.007). The rest of the details are shown in Table 3 below.

**Table 3:** The mean Egg load (EPG) of soil transmitted helminths infections among the 562 preschool-age children that participated in the study in Hoima District

Parasite	Arithmetic Mean EPG	Range	Geometric mean EPG	95% CI
Hookworm				
Overall	1675.2	(48–13,200)	696.1	(530.3–913.8)
Male	1396.7	(48–5280)	539.0	(446.9–913.6)
Female	2086.3	(120–13,200)	789.8	(511.6–1219.3)
Age group				
1–2 years	981.9	(72–4200)	482.9	(281.6–828.3)
3–4 years	1679.5	(48–10,800)	689.3	(456.4–1041.0)
5 years	2199.2	(48–13,200)	936.9	(562.3 - 1560.9)
A. lumbricoides				
Overall	1118.4	(120–7200)	569.7	(418.7–775.3)
Male	1310.2	(120–7200)	663.6	(436.5–1009.0)
Female	870.5	(120–3360)	467.9	(290.5–753.5)
Age group				
1–2 years	1417.7	(129–7200)	634.5	(293.5–1371.3)
3–4 years	1250.8	(120–7200)	680.5	(430.2 - 1076.4)
5 years	660.0	(120–2400)	391.1	(229.1–667.7)
T. trichiura				
Overall	784	(168 - 1920)	440.0	(17.6–11,001.6)
Male	168	–	168.0	–
Female	1092	(264–1920)	712.0	(0.0–6319.3)
Age group				
1–2 years	1044	(168–1920)	567.9	(0.0–24,228.2)
3–4 years	–	–	–	–
5 years	264	–	264.0	–

**Fig. 3:**
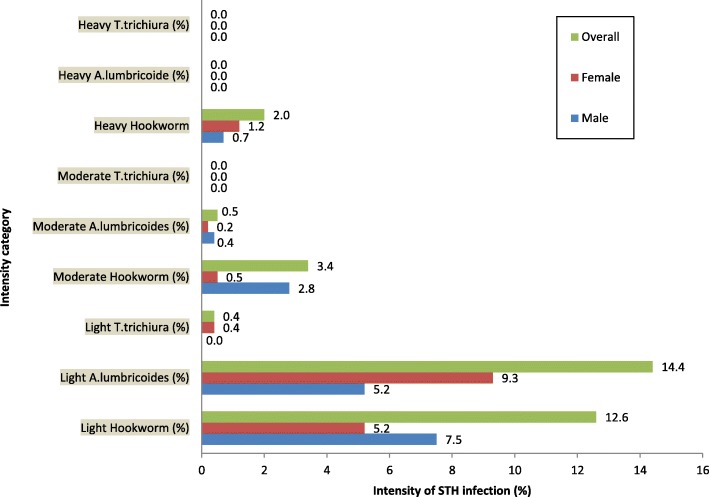
Intensity of STH infection among the 562 PSAC that participated in the study in Hoima District

Of the children whose stool samples were positive for Hookworm infection, 12.6% (71/562) had light (1–1999 EPG) intensity, 3.4% (19/562) had moderate (2000–3999 EPG) and 2.0% (11/562) had heavy (> = 4000 EPG) infections intensity. As for *A. lumbricoides* infections, 14.4% (81/562) had light (1–4999epg) infection intensity, 0.5% (3/562) had moderate (5000–49,999 EPG) infection intensity. The proportions of children with light (1–999 EPG) and moderate (1000–9999 EPG) *T. trichiura* infection were 0.4% (2/562) and 0.2% (1/562), respectively. All the participating children had no heavy *A. lumbricoides* and *T. trichiura* infections, according to WHO classification [38]. The rest of the details are shown in Fig. 3 below.

### Factors associated with STH infections among preschool-age children

Additional file 4 shows the distribution of background and socio-behavioural factors associated with STH infections among PSAC. Age had significant association with STH infection (*p* = 0.002) that PSAC aged 5 years were 1.2 times more likely (adj.PR = 1.2, 95% CI: 1.2–2.6) to be infected than those aged 1–2 years. After adjusting for gender, handwashing before eating and after defecation, sandal wearing, meal preparation, eating raw/uncooked vegetable, eating unwashed fruits, place of residence, toilet facility, human/domestic wastes disposal, SES, caretaker’s education, knowledge about STH infection and child de-worming. The odds of infection were 2.6 times higher (adj.PR = 2.6, 95% CI: 1.9–3.6) for children who rarely wash hands after defecation compared to those who often wash hands after defecation. Children who were reported to have eaten raw or uncooked vegetables were 1.9 time more affected (adj. PR = 1.9, 95% CI: 1.3–2.7) than those who eat cooked vegetables. The odds of infection were 1.8 times higher (adj. PR = 1.8, 95% CI: 1.1–2.8) for children eating fruits without washing or peeling them off as compared to children who wash or peel off before eating fruits. Children who did not swallow deworming drugs for STH were 1.4 times at higher risk (adj. PR = 1.4, 95% CI: 1.0–1.8) of being infected with STH as compared to those who swallow the drugs As expected, children from families that practiced unsanitary disposal of faeces were 1.5 times more likely (adj.PR = 1.5, 95% CI: 1.1–2.0) to be infected with STH than those from families that practice sanitary disposal,after adjusting for the effects of other significant factors in the model. The other considered socio-demographic and economic factors didn’t show significant level of association with STH infection (Additional file 4).

After adjusting for the effects of other significant factors in the model and potentially confounding and/or modifying variables, we found that older age, not handwashing with soap after defecation, eating vegetables and fruits without washing them, improper disposal of youngest child faeces, having caretakers with tertiary education and not deworming PSAC regularly were statistically and independently associated high risk for infections with STH. On the other hand, wearing sandal most of time while outside the home, having children’s food prepared by a person aged 20 years above, living in upland area, using pit latrine for defecation, moderate knowledge about STH infection and belonging to a family of highest socio-economic status were statistically and independently associated with lower risk of STH infection among PSAC.

## Discussion

The overall prevalence of STH infection in PSAC was 26.5%, which is higher than the estimated prevalence of 9% among the PSAC in Hoima district. This is not surprising and it shows that community survey may be more effective in identifying PSAC with STH infections than facility-based secondary data [6, 23, 24]. The overall prevalence of the current study was also higher than the prevalences of 19.7, 23.3 and 24.3% reported in studies conducted in Dschang (Cameroon), Butajiri (Ethiopia) and Wonji Shoa sugar estate (Ethiopia) [15, 39, 40]. However, it is lower than the results of studies conducted in Sheska Kekele (Ethiopia) and Osun State (Nigeria) [41, 42]. The differences in the prevalence and distribution of STH among different communities might be due to differences in environmental conditions and poor knowledge and awareness about STH [15, 43]. For instance, areas with poor drainage system may experience frequent flooding and surface run-off during rainy seasons. This may lead to contamination of vegetables and fruits gardens and water sources especially in areas with poor human and domestic waste disposal system. Such conditions expose the children to contaminated soil, thus increasing their susceptibility to STH infection [36, 44].

In the present study, Hookworm was the most common species of STH recovered from the children. This is consistent with the findings of similar studies conducted in other settings [43, 45]. In rural settings where children walk bare foot, Hookworm infection may be more prevalent than *A. lumbricoides* and *T.trichiura* [30]. Other studies have reported *T.trichiura* as the predominant STH among PSAC [41, 46, 47]. These areas have cold climatic conditions, more favourable for the survival of *T.trichiuris* [44]. Many endemic countries have designed and implemented short deworming treatment intervals (3-monthly) with Albendazole and praziquantel combined with health education on personal and environmental hygiene so as to reduce the prevalence of intestinal helminths among children. Currently, Uganda is implementing four-monthly antihelminthic treatments with albendazole deworming program under the child health day plus strategy, which started in 2004. However, the effectiveness of the control efforts remain difficult to measure because of lack of routine monitoring, surveillance and periodic program evaluation [1, 20].

Although our study focused on the preschool-age children, the 26.5% prevalence reported is higher than the 17.2% prevalence that Standley and colleagues reported in their study of intenstinal schistosomiasis and STH infections among school going children in Lake victoria Region of Uganda [48]. This clearly demonstrates that STH infection is still a public health problem not only among school-age children but also among PSAC. Previous studies have revealed that moderate to heavy SHT infection could result in chronic dysentery, iron deficiency anaemia, intestinal obstruction, reduced physical growth and cognitive development [15, 49, 50]. These studies reported light and moderate STH infections among PSAC, which is similar to the results of our finding. This therefore suggest that PSAC continue to suffer from heavy infection intensity, which eventually leads to long term physical and mental growth retardations.

In this study, females had higher mean egg intensity of Hookworm infection than males. Other studies have congruously demonstrated that females have indeed heavy worm burdens, compared to their male counterpart [42, 51]. However, the cause of this in PSAC is yet unknown [42, 52] and could be attributed to behavioural differences between males and females [42, 52]. A study conducted in South Africa found a strong association between soil eating and Ascaris infection among school-age females. There is need to conduct further studies to explore factors that may explain observed gender differences in worm specific infection intensity among PSAC in these study areas.

The results of our findings showed that the likelihood of being infected with STH is significantly higher in children aged 5 years than in children aged 1–2 year. This is in conformity with the findings from other studies [15, 53, 54]. This is not surprising because in rural settings, hygiene and sanitation conditions are normally poor, yet it is allowed for older PSAC to interact with other children in the neighborhood. Their playful behaviours expose them to contaminated soil, food and/or water, hence increasing the risk of re-infection [4, 39, 55].

Studies conducted in Lake Victoria Region of Uganda and Western Côte d’Ivoire revealed that lack of funding for logistics and community mobilization in hard to reach areas hampers efforts to increase uptake of deworming treatment [7, 48, 56]. The results from our study showed that PSAC living in lowland residential areas were associated with higher likelihood of being infected with STH than those in upland areas. This suggests that apart from unhygienic conditions they face, many children in lowland and hard to reach areas may increasingly be exposed to infections mainly because some of them miss out deworming treatment scheduled for April and October of every year. In the rural context, caretakers may be busy preparing their gardens for cultivation as well as harvesting crops during the months of April and October respectively. This may contribute to forgetting deworming program schedule unless they are regularly reminded using Information, Education and Communication (IEC) materials. Another reason for missing out deworming treatment could be attributed to health workers’ failure to reach children with the deworming services because of logistical challenges.

Although the likelihood of acquiring STH infection was higher among children of caretakers with tertiary education than those whose caretakers had no formal education, the number is small (tertiary education 41) compared to other groups. Strikingly, the actual prevalence is lower in the tertiary group compared to the no formal education group (24.4 to 38.9%). This is surprising and it suggests that while the number of highly educated caretakers in the rural communities is relatively small, most of them may spend more time in outdoor activities as they fend for their families than they have time with their children. In their absence, teenagers who may maintain poor personal and food hygiene are forced to take care of the young ones, hence increasing PSAC’s susceptibility to infection. This argument is supported by the results of our finding that in homes where meals (breakfast and lunch) for young children are normally prepared by persons aged < 15 years, the risk of STH infection is higher than in homes where meals are normally prepared by persons aged ≥20 years. Further research is needed to explore the reasons: “Why children from families of caretakers with tertiary education tend to be at higher risk for STH infections than children of caretakers with no formal education?”

The results of this study revealed that personal hygiene such as clean hands and sandals wearing play important role in transmission of STH infections among the PSAC. This is similar to the findings of other studies [31, 43, 53]. In poor rural communities, there is high tendency for children to walk barefoot and to interact with contaminated soil, water and objects, thus increasingly exposing them to different forms of infections including STH [57, 58]. Sandal wearing and hand washing with soap after defecation and before eating food are some of the preventive measures against STH. However, unless caretakers of children understand that the larvae/eggs of parasitic worms can be transmitted through soil, skin and ingestion of dirty food and water, efforts to reduce the prevalence and burden of STH infection may be frustrated. There is need to increase people’s awareness on the importance of proper personal and environmental hygiene particularly in communities with poor water, sanitation and hygiene. This study also affirms that eating unwashed fruits and uncooked vegetable were positively associated STH infection, which is similar to what was reported in other studies [49, 53]. This could imply lack of knowledge about transmission and prevention of STH [39, 58].

In this study, open defecation, unsanitary disposal of faeces of young children and inadequate knowledge about transmission and prevention of STH were factors associated with STH infections among PSAC. This is similar to what other studies have reported [16, 39, 45, 59]. This implies that in situations where human and animal wastes are improperly disposed, the level of transmission of the infective eggs of parasitic worm is high [45]. It could also imply poor living standard and low level of education of caretakers, which may lead to poor access to safe water supply and improper human, animal and domestic wastes disposal system. Using self reports to factors associated with STH infections are also noted in the current study. However, we controlled it by using validated tools. There were some limitations in this study; firstly, anticipated higher prevalence of A. *lumbricoides* and T*.trichiuris* infections than what was reported in the current study if we had conducted a study during the rainy season of the year (June–August 2016). During this period children may increasingly be exposed to contaminations as they eat a lot of fruits and vegetables. Secondly, the reasons as to why girls presented with higher Hookworm intensity than boys and why some children did not receive de-worming treatment, could have been established through qualitative survey in the study area, thirdly, the average time taken to deliver stool samples for examinations was 4 h. Therefore, adding formol-ether with a delay of more than 4 h could have reduced our ability to detect more Hookworm infection and fourthly, we expected that more children would be diagnosed with STH infection if we had collected more than one stool sample per child on consecutive days. However, we increased chance of diagnosing STH by increasing the number of slide per sample during stool examinations. We also used experienced laboratory technicians to independently validate results by re-examining sample slides. There is need to conduct further studies to explore the reasons and draw strong conclusion about gender differences in worm specific infection intensity among PSAC in the study areas.

## Conclusion

The prevalence of Soil transmitted helminths infection among preschool-age children in Hoima district was 26.5%. Hookworm infection intensity was the highest followed by *A. lumbricoides* and the mean egg intensity was higher among older children than younger ones. Not washing hands with soap after defecation, eating vegetables and fruits without proper cooking and or washing them, improper disposal of faeces of young children and irregular deworming of preschool-age PSAC are some of the factors associated with STH infection. There is need to create awareness about latrine construction and utilization as well as the importance of keeping personal and environmental hygiene particularly in communities with poor Water Sanitation and Hygiene (WASH). Deworming programs should focus on reaching communities in lowland areas, in addition to exploring the feasibility and cost effectiveness of shortening deworming treatment intervals from 6-monthly to 4-monthly, in order to reduce the risk of STH infections among PSAC.

## Additional files

Additional file 1: Prevalence of soil transmitted helminths infections among the 562 preschool-age children that participated in the study in Hoima. (XLSX 18 kb)

Additional file 2: Contains data used to draw Fig. 1 (The Prevalence of STH), Fig. 2 (the distribution of STH and the intensity of STH among the 562 PSAC in Hoima district) & Fig. 3 (Intensity of STH infection among the 562 PSAC that participated in the study in Hoima District). (XLSX 18 kb)

Additional file 3: Contains data used to draw conclusions. (XLSX 67 kb)

Additional file 4: Factors associated with Soil-transmitted helminths infections among the 562 preschool-age children that participated in the study in Hoima District. (XLSX 13 kb)

## Abbreviations

CI: Confidence interval
EPG: Eggs per gram of stool
EPI: Expanded program on immunization
GM: Geometric mean
IEC: Information, education and communication
JMP: Joint monitoring programme
PCA: Principal component analysis
PR: Prevalence ratio
PSAC: Preschool-age children
SD: Standard deviation
SES: Socio-economic status
STH: Soil-transmitted helminthiasis
UNICEF: United Nations International Children Education Funds
WASH: Water sanitation and hygiene
WHO: World Health Organization
